# Comparative Analysis of Biologically Relevant Response Curves in Gene Expression Experiments: Heteromorphy, Heterochrony, and Heterometry

**DOI:** 10.3390/microarrays3010039

**Published:** 2014-02-14

**Authors:** Stuart G. Baker

**Affiliations:** Biometry Research Group, National Cancer Institute, Bethesda, MD 20872, USA; E-Mail: sb16i@nih.gov

**Keywords:** double sigmoid, microarray, relative prediction error, sigmoid, time series

## Abstract

To gain biological insights, investigators sometimes compare sequences of gene expression measurements under two scenarios (such as two drugs or species). For this situation, we developed an algorithm to fit, identify, and compare biologically relevant response curves in terms of heteromorphy (different curves), heterochrony (different transition times), and heterometry (different magnitudes). The curves are flat, linear, sigmoid, hockey-stick (sigmoid missing a steady state), transient (sigmoid missing two steady states), impulse (with peak or trough), step (with intermediate-level plateau), impulse+ (impulse with an extra parameter), step+ (step with an extra parameter), further characterized by upward or downward trend. To reduce overfitting, we fit the curves to every other response, evaluated the fit in the remaining responses, and identified the most parsimonious curves that yielded a good fit. We measured goodness of fit using a statistic comparable over different genes, namely the square root of the mean squared prediction error as a percentage of the range of responses, which we call the relative prediction error (RPE). We illustrated the algorithm using data on gene expression at 14 times in the embryonic development in two species of frogs. Software written in Mathematica is freely available.

## 1. Introduction

Many gene expression experiments involve serial measurements in response to a varying condition, such as temperature, oxygen availability, time, drug concentration, levels of pollutants, and exposures to ultraviolet light. Often investigators want to compare the time varying response between two scenarios, such as two species or two drugs. For this comparative analysis, we developed an algorithm to fit biologically relevant curves to serial response measurements from each gene, identify pairs of curves that fit well, and compare these curves under the two scenarios in terms of heteromorphy (different curves), heterochrony (different transition times) and heterometry (different magnitudes). In the context of ontogeny, Yanai *et al*. [1] introduced the concepts of heteromorphy and heterochrony in gene expression curves as analogs to tissue-level heteromorphy (different sizes of developing organs) and heterochrony (movement of modules in anatomy and physiology). In other comparative gene expression settings heteromorphy and heterochrony in gene expression curves can also provide insight into biological processes. The purpose of this methodology is to compare gene expression patterns in two settings, as guided by biologically relevant models. To simplify this discussion, we consider time as the time varying condition.

The fitting part of the algorithm involves the following models: flat, linear, sigmoid, double sigmoid [2], and generalized double sigmoid [3]. The flat and linear models yield flat and linear response curves, respectively. The sigmoid model yields a sigmoid curve (two steady states with an intermediate transition following a logistic function), a hockey stick curve (sigmoid curve missing one steady state) or a transition curve (sigmoid missing two steady states). The double sigmoid is the product of two sigmoid models; it yields an impulse curve (with a peak or trough) or step curve (with an intermediate-level plateau). The generalized double sigmoid curve adds an additional parameter to the double sigmoid model and yields analogous impulse+ curve or step+ curves. We also characterized all the curves, except for flat, as either trending upward or downward or having a downward or upward impulse. 

The aforementioned response curves are biologically relevant, as opposed to polynomial curves of degree two or greater, which generally have little biological basis. Flat curves represent a steady state. Linear curves represent the constant addition or subtraction of reacting components. Sigmoid curves model the addition or subtraction of reacting components from one steady state to another steady state. Sigmoid curves also arise in transcription factor binding [4,5]. Impulse and impulse+ curves represent a temporary increase or decrease in reacting components that resolves into a new steady state [1]. Step and step+ curves represent an intermediate-level steady state amid a monotonically increasing or decreasing number of reacting components. 

Although there is a large literature on the fitting of response curves to sequential gene expression measurements in dose-response and short time series studies [6,7,8,9,10], there has been little work on the comparative analysis of response curves. A notable exception is Sivriver, *et al.* [3] who fit and compared generalized double sigmoid response curves under two stimuli. A major concern of Sivriver *et al.* [3] was overfitting. Overfitting means that a model has so many parameters relative to time points that chance deviations from the true model strongly influence the model fit and lead to poor predictions at time points not used in model fitting. Here overfitting is particularly a concern for two reasons. First the generalized double sigmoid and double sigmoid models have a large number of parameters relative to the number of time points. Second the investigation of over ten thousands genes implies a much higher probability of substantial chance deviations in the response curves for some genes than if only a few genes were studied. Sivriver, *et al.* [3] tackled the problem of overfitting by pooling data from multiple genes with similar generalized double sigmoid response curves. Because we focus on heterochrony and heterometry, which would be diluted by pooling, we developed a different approach to reduce overfitting that does not involve pooling. In our approach we evaluated model fits at different times from those used to fit the model. In particular we fit biologically relevant curves to every other response (first, third, fifth, …) and evaluated the fits at the remaining responses (second, fourth, sixth, …), providing an empirical investigation of model fit. Because we used seven points for model fitting and seven points for model evaluation, we needed at least 14 points to adequately fit and evaluate the generalized double sigmoid model, which has seven parameters. 

For illustration, we applied our algorithm to mean gene expression levels (averaged over three technical replicates and three specimens) for 11,299 genes at 14 development times in two species of frogs, *X.laevis* and *X.tropicalis.* [1]. We found some interesting examples of heteromorphy and heterochrony that will hopefully spur new research. However, the main contribution of this paper is a method for identifying the most interesting changes in pairs of biologically relevant shapes for gene expression curves in comparative studies. 

## 2. Identifying Biologically Relevant Response Curves that Fit Well

As noted by Forster [11] standard methods of model selection (such as likelihood ratio tests, the Akaike Information Criterion (AIC), the Bayesian Information Criterion, and Minimum Description Length [11,12,13]) minimize the discrepancy between predicted and observed results at the *same* time points used to fit the model. In the spirit of Forster [11] and with the emphasis on reducing overfitting, we were instead interested in minimizing the discrepancy between predicted and observed results at *different* time points than used to fit the model. Similarly, Chechik and Koller [2] evaluated double sigmoid fits at a single point that was left out of the fitting procedure. We considered every other point as left-out in order to examine discrepancy between observed and predicted results over the entire range of times.

Consider a single gene. Let *y_j_* denote the *j*^th^ observed response and *x_j_* denote the *j*^th^ observed time. We fit the model to responses {*y_1_*, *y_3_*, *y_5_*, *y_7_*, *y_9_*, *y_11_*, *y_13_*} at times {*x_1_*, *x_3_*, *x_5_*, *x_7_*, *x_9_*, *x_11_*, *x_13_*}. We call {(*x_1_*, *y_1_*), (*x_3_*, *y_3_*), (*x_5_*, *y_5_*), (*x_7_*, *y_7_*), (*x_9_*, *y_9_*), (*x_11_*, *y_11_*)} the *fitted points.* We evaluate the model at {(*x_2_*, *y_2_*), (*x_4_*, *y_4_*), (*x_6_*, *y_6_*), (*x_8_*, *y_8_*), (*x_10_*, *y_10_*), (*x_12_*, *y_12_*)}, which we call the *evaluation points* Let {*f*(*x_2_*), *f*(*x_4_*), *f*(*x_6_*), *f*(*x_8_*), *f*(*x_10_*), *f*(*x_12_*), *f*(*x_14_*)}denote the predicted responses of a particular model corresponding to the evaluation points. 

We needed a measure of how well the predicted responses fit the observed evaluation points. One measure considered was the mean squared error (MSE). The problem with using MSE is that it depends on the absolute sizes of responses, so two genes could have the same MSE’s for comparing predicted and observed responses, yet visually one may fit well and the other fit poorly. To circumvent this problem we introduced the Relative Prediction Error (RPE), which is the square root of the MSE of the predicted response divided by the difference between the largest and smallest predicted responses, expressed as a percentage. The reason for using the square root is to put the measure on the same scale as the responses, analogous to using a standard deviation instead of a variance. The reason for dividing by the range of responses is to make small deviations between predicted and observed response relative to the entire shape of the curve, which leads to a visually satisfying measure. Let *J =*{2, 4, 6, 8, 10, 12, 14} index the evaluation points. Mathematically we write RPE for our situation with 14 time points as

RPE = [Σ*_j in J_* {*y_j_* − *f(x_j_*)}^2^/7]^1/2^/[max*_j in J_* {*f*(*x_j_*)} − min*_j in J_* {*f*(*x_j_*)}] (1)

The formula for RPE can be readily modified for more than 14 points. Based on a visual inspection of curves with different values of RPE, we decided that a threshold of 10% was a reasonable indicator of a good fit. To put the idea of a threshold RPE into perspective, note that a likelihood ratio test comparing observed and fitted counts typically also involves a threshold, namely a 5% type I error. 

When comparing predicted and observed results at *different* time points than used to fit the model, Forster [11] evaluated the fit of the model without considering the complexity of the model. A rationale is that an evaluation at different time points than used for fitting inherently penalizes for complexity that leads to overfitting. Nevertheless, visual inspection suggests that parsimony is desirable for characterizing curves based on their fits to the evaluation points. For characterizing parsimony using the evaluation points we allow a 5% leeway in terms of RPE for a curve with fewer parameters than the curve with smallest RPE. In other words, if a response curve has fewer parameters than the response curve with smallest RPE, we prefer the response curve with fewer parameters if its RPE is less than or equal to the smallest RPE plus 5% We chose the value of 5% based on visual inspection of many curves.

To introduce the curve selection algorithm, consider the following two hypothetical examples for a single gene. In the first example, suppose the RPE’s for flat, lineU, sigmoidU, impulseU, and impulse+U curves are 30%, 12%, 11%, 8%, and 9%, respectively (as explained in the next section, the “U” designates upward trend). 

*Step 1.* In this first example the best fitting curve is impulseU because it has the smallest RPE, namely 8%. Because this RPE of 8% is less than or equal to the 10% RPE threshold for a good fit, we consider impulseU a good fit and investigate a more parsimonious curve in *Step 2*. Otherwise, if this RPE were greater than 10%, we would not report a response curve for this gene. 

*Step 2.* In this first example, lineU and sigmoidU satisfy the 5% RPE leeway requirement, both having an RPE ≤ 8% + 5% = 13%. Because lineU has fewer parameters than sigmoidU, we select lineU as the reported response curve. 

In the second hypothetical example, suppose the RPE’s for flat, lineU, sigmoidU, impulseU, and impulse+U curves are 30%, 22%, 14%, 8%, 9%, respectively.

*Step 1.* In this second example, the best fitting curve is impulseU because it has the smallest RPE, namely 8%. Because it is a good fit with RPE < 10%, we investigate a more parsimonious model in *Step 2*.

*Step 2.* In this second example, no curve with fewer parameters than impulseU satisfies the 5% RPE leeway requirement. Therefore we select impulseU as the reported response curve. However, for purposes of comparison, we identify the curve with the next fewest parameters than impulseU, namely sigmoidU. 

We formalize the curve selection algorithm as follows. 

*Step 1.* Let Curve A denote the response curve with the smallest RPE, which we denote RPE_A_. In the first example Curve A is impulseU. If RPE_A_ > 10%, report no curve; otherwise proceed t*o Step 2.*

*Step 2.* We identify a Curve B as follows. Let CurveSet_B_ denote the set of response curves with fewer parameters than Curve A. In the first example CurveSet_B_ = {flat, lineU, sigmoidU}. Let CurveSubset_B_ denote a subset of response curves in CurveSet_B_ such that RPE ≤ RPE_A_ + 5%. In the first example CurveSubset_B_ is {lineU, sigmoidU}. If CurveSubset_B_ is the empty set, we identify Curve B as the curve with the most parameters in CurveSet_B_ (sigmoidU in the second example) but select Curve A as the reported curve. If CurveSubset_B_ is not empty we identify Curve B as the curve in CurveSubset_B_ with the fewest parameters (lineU in the first example) and select Curve B as the reported curve. 

When we report a pair of response curves for a gene, we require that each response curve in the pair yield a good fit to the data with RPE_A_ ≤ 10%. The curve reported for each gene in the pair is either Curve A or Curve B, whichever was selected via the curve selection algorithm.

**Figure 1:**
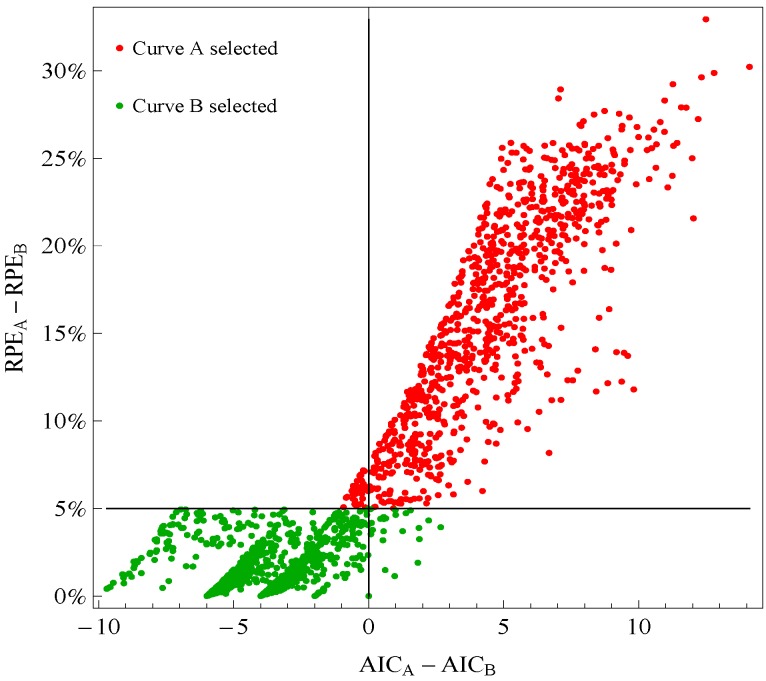
Comparison of a change in relative prediction error (RPE) with a change in Akaike Information Criterion (AIC) among response curve pairs. The red points corresponding to Curve A require RPE_A_− RPE_B_ ≤ 5% (so are above the horizontal 5% line) The green points corresponding to Curve B require RPE_A_ − RPE_B_ ≤ 5% (so are below the horizontal 5% line). A value of AIC_A_ − AIC_B_ ≤ 0 (so on the left of vertical line) would indicate selection of Curve B.

In our application to frog data, the 5% RPE leeway agreed well with the sign of the change in AIC, where AIC = 7 log [Σ*_j in J_* {*y_j_* − *f(x_j_*)}^2^/7] + 2 × (number of parameters). Although this is a non-standard use of AIC because it applies to evaluation points instead of fitted points, it is still instructive. Figure 1 plots points for genes with good fitting models in both species of frogs. The points labeled Curve A (Curve B) selected correspond to reporting Curve A (Curve B) in the curve selection algorithm. Most Curve A selected points, which require RPE_A_ − RPE_B_ > 5%, correspond to AIC_A_ − AIC_B_ > 0 (the upper right quadrant). Most Curve B selected points, which require RPE_A_ − RPE_B_ ≤ 5%, correspond to AIC_A_ − AIC_B_ ≤ 0 (the lower left quadrant). 

## 3. Fitting Algorithms

We fit all models using iteratively reweighted least squares with modifications to incorporate starting values. Let *x* denote the varying condition, such as time. We discuss the formulas and fitting of each biologically relevant response curves in turn.

### 3.1. Flat

The flat curve has equation f_FLA_(*x*) = *α*_FLA_. 

### 3.2. Linear

The linear curve has equation f_LIN_(*x*) = *α*_LIN_ + *β*_LIN_·*x*, for *β*_LIN_ ≠ 0. Letting b_LIN_ denote the estimate of *β*_LIN_, we designated the linear model as lineD if b_LIN_ < 0 and lineU if b_LIN_ > 0, where D stands for downward and U stands for upward. 

### 3.3. Sigmoid

The sigmoid curve starts with a steady state and then monotonically increases or decreases and finishes with another steady state (Figure 1). For flexibility, we fit one of two versions of the sigmoid model, depending on the estimated slope of the linear model,

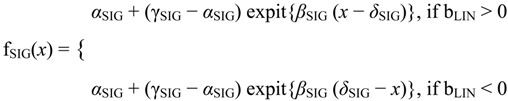
(2)
where expit(*x*) = exp(*x*)/{1 + exp(*x*)}. The parameters *α*_SIG_ and γ_SIG_ specify levels of the steady states. The parameter *δ*_SIG_ is the horizontal point corresponding to the maximum slope, *β*_SIG_, between the steady states. The sign of b_LIN_ is not always a reliable guide to the trend of the sigmoid curve, which we determine by simply comparing the first and last points on the sigmoid curve. We designated the downward and upward trending sigmoid curves as sigmoidD and sigmoidU, respectively.

### 3.4. Hockey Stick

The hockey stick curve is a sigmoid curve that is missing one steady state. We identified a steady state in a sigmoid curve as a slope at the beginning or the end of the curve that is less than or equal to 0.10, a value chosen based on visual inspection. We designated the downward and upward trending hockey-stick curves as hockeyD and hockeyU, respectively.

### 3.5. Transition

A transition curve is a sigmoid curve that is missing two steady states, leaving only the transition region between the missing steady states. We designated the downward and upward trending transition curves as transitionD and transitionU, respectively.

### 3.6. Impulse

The impulse curve is one type of curve (along with the sigmoid and step curves) arising from the double sigmoid model. For flexibility, we fit one of two versions of the double sigmoid model, depending on the estimated slope of the linear model,

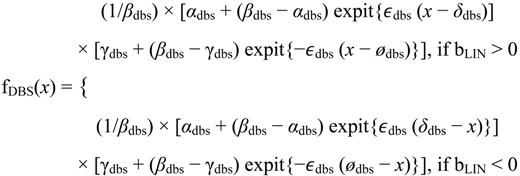
(3)


To avoid numerical problems, we only fit the double sigmoid model if the RPE of the sigmoid model was larger than the RPE of the linear model. Starting values come from the fit of the sigmoid model, namely *α*_dbs_ = *a*_sig_, *β*_dbs_ = (*a*_sig_ + *g*_sig_)/2, γ_dbs_ = *g*_sig_, *δ*_dbs_ = *δ*_sig_ , *ϵ*_dbs_ = 0, and *ø*_dbs_ = 0, where *a*_sig_ and *g*_sig_ are the estimates of α_sig_ and γ_sig_, respectively.

**Figure 2:**
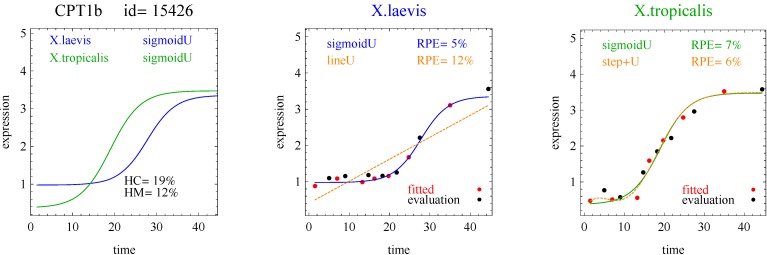
Example of sigmoid curves for one gene pair. *X.laevis* and *X.tropicalis* are two species of frogs. The red points denoted “fitted” were used for model fitting. The black points denoted “evaluation” were used for model evaluation and computation of RPE. The curves with blue and green labels and lines are the reported curves (Curve A or Curve B, whichever was selected). The curves with orange labels and lines are included for comparison (Curve A or Curve B, whichever was not selected). HC and HM are measures of heterochrony and heterometry, respectively.

The impulse curve has a peak or trough between steady states (Figure 2) although sometimes the steady states are missing. The parameters α_dbs_ and γ_dbs_ correspond to levels of the flat sections. For example with *b_LIN_* > 0 and *ϵ*_dbs_ > 0, f_DBS_(*x*) is approximately (1/*β*_dbs_) × *α*_dbs_ × *β*_dbs_ = *α*_dbs_ for small values of *x* and approximately (1/*β*_dbs_) × *β*_dbs_ × γ_dbs_ = γ_dbs_ for large values of *x.* The parameter *β*_dbs_ determines the level of the impulse. The parameter *ϵ*_dbs_, which appears in each sigmoid factor, determines the slope of the peak or trough. Mathematically, we identified the impulse curve as a double sigmoid curve in which the minimum or maximum did not occur at the endpoints. We designated an impulse curve with a trough and peak as impulseD and impulseU, respectively.

### 3.7. Step

The step curve is a double sigmoid curve with an intermediate plateau between steady states, although sometimes the steady states are missing. Mathematically, we identified the step curve as a double sigmoid curve in which both the minimum and maximum occur at the endpoints. Although this identification procedure would also detect a sigmoid curve, the sigmoid curve is preferentially selected via the sigmoid model. HoHWe designated the downward and upward trending step curves as stepD and stepU, respectively.

### 3.8. Impulse+

Sivriver, *et al.* [2] generalized the impulse double sigmoid model to allow for different slopes before and after the peak or trough of an impulse curve. We call the analog of the impulse curve for the generalized double sigmoid model the impulse+ curve. We parameterized the generalized double sigmoid by multiplying −*ϵ*_dbs_ in Equation (3) by an additional parameter λ_dbs_. We used the parameter estimates from the impulse curve as starting values with λ_dbs_ = 0. We identified the impulse+ curve as a generalized double sigmoid curve in which the minimum or maximum was not at the endpoints. We designated an impulse+ curve with a trough and peak as impulse+D and impulse+U, respectively.

### 3.9. Step+

We identified the step+ curve as a generalized double sigmoid curve in which both the minimum and maximum occur at the endpoints. We designated the downward and upward trending step+ curves as step+D and step+U, respectively. 

## 4. Measuring Heterometry and Heterochrony

### 4.1. Heterometry

We measured heterometry (HM) as the mean vertical difference between response curves expressed as a percentage of the vertical response range. We computed HM based on the following points on the response curves: (i) any point on the flat curve; (ii) the endpoints for linear, transient, hockey, sigmoid, or step and step+, curves; and (iii) the endpoints and the point at the peak or trough for impulse and impulse+ curves. For Table 1, our indicator of heterometry was HM ≤ 10%. 

**Table 1:** Response curve pairs with at least five counts.

*X.laevis*	*X.tropicalis*	Total	Heterochrony only	Heterometry Only	Heterochrony and heterometry
sigmoidU	sigmoidU	694	18	347	70
lineU	sigmoidU	146	0	0	0
sigmoidU	hockeyU	73	0	0	0
sigmoidD	sigmoidD	48	3	22	0
lineU	lineU	47	0	39	0
hockeyU	hockeyU	30	0	18	1
sigmoidU	lineU	20	0	0	0
lineU	hockeyU	14	0	0	0
hockeyU	sigmoidU	13	0	0	0
sigmoidU	impulseD	9	0	0	0
impulseD	sigmoidU	8	0	0	0
sigmoidD	lineD	8	0	0	0
sigmoidD	hockeyD	5	0	0	0

### 4.2. Heterochrony

We measured heterochrony (HC) as the mean horizontal difference between response curves as a percentage of the horizontal response range. We computed HC based on the following points on the response curves: (i) the horizontal point at the maximum absolute value of slope for sigmoid, hockey, transition, step, and step+ curves; and (ii) the horizontal point corresponding to the peak or trough of the impulse and impulse+ curves. We did not compute HC for flat or linear curves. For Table 1, our indicator of heterochrony was HC ≤ 10%.

## 5. Results

**Figure 3:**
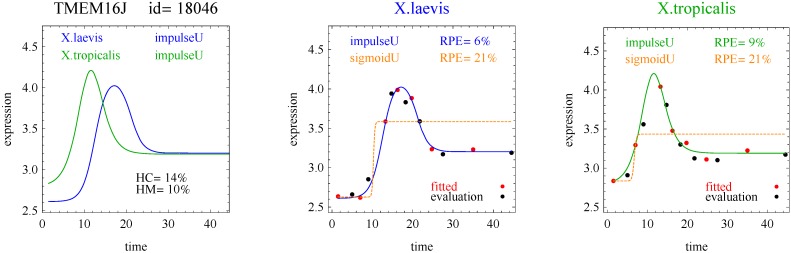
Example of impulse curves for one gene pair.

**Figure 4:**
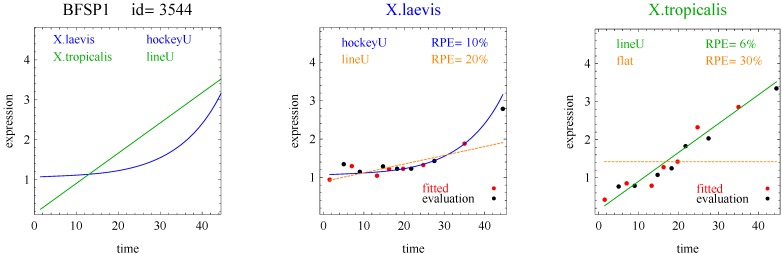
Example of a hockey stick and line for one gene pair.

**Figure 5:**
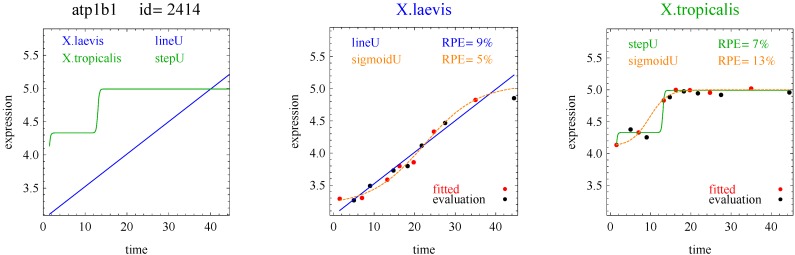
Example of a step curve and line for one gene pair.

Of the 11,299 genes in the frog data, 10% of the response curves were good fits in both species of frogs (and used for the analysis) and 45% were good fits in only one species of frog. Table 1 shows the distribution of the reported response curve pairs with at least five counts. The sigmoid curve predominated along with the closely related hockey-stick curve, but with relatively few impulse curves. Importantly, the algorithm did not select any transition, impulse+, or step+ curves as best fitting with good fits to the data. The predominance of heterometry of heterochrony in Table 1 confirms earlier less formal investigations [1]. By examining lists of genes corresponding to the model pairs with nonzero counts in Table 2, Table 3, Table 4 and Table 5 and inspecting the fits with figures like Figure 2, Figure 3, Figure 4 and Figure 5, investigators may be able to gain more insight into differences between the development of *X.laevis* and *X.tropicalis*. Of particular note are examples of heteromorphy in which the pair of curves trended in opposite directions (Table 3 and Table 4). 

**Table 2:** Counts for response curve pairs with downward trends for both *X.laevis* and *X.tropicalis.* The total number is 68.

	*X.tropicalis*								
***X.laevis***	flat	lineD	tranD	hocD	sigD	impD	stepD	imp+D	step+D
Flat	0	0	0	0	0	0	0	0	0
lineD	0	3	0	0	1	0	0	0	0
tranD	0	0	0	0	0	0	0	0	0
hocD	0	0	0	0	0	0	0	0	0
sigD	0	8	0	5	48	1	0	0	0
impD	0	0	0	0	0	2	0	0	0
stepD	0	0	0	0	0	0	0	0	0
impD+	0	0	0	0	0	0	0	0	0
stepD+	0	0	0	0	0	0	0	0	0

**Table 3:** Counts for response curve pairs with downward trends for *X.laevis* and upward trends for *X.tropicalis.* The total number is 14.

	*X.tropicalis*								
***X.laevis***	flat	lineU	tranU	hocU	sigU	impU	stepU	imp+U	step+U
flat	0	0	0	0	0	0	0	0	0
lineD	0	0	0	0	0	0	0	0	0
tranD	0	0	0	0	0	0	0	0	0
hocD	0	0	0	0	0	0	0	0	0
sigD	0	0	0	1	1	0	0	0	0
impD	0	4	0	0	8	0	0	0	0
stepD	0	0	0	0	0	0	0	0	0
impD+	0	0	0	0	0	0	0	0	0
stepD+	0	0	0	0	0	0	0	0	0

**Table 4:** Counts for response curve pairs with upward trends for *X.laevis* and downward trends curves for *X.tropicalis.* The total number is 16.

	*X.tropicalis*								
***X.laevis***	flat	lineD	tranD	hocD	sigD	impD	stepD	imp+D	step+D
flat	0	0	0	0	0	0	0	0	0
lineU	0	0	0	0	1	2	0	0	0
tranU	0	0	0	0	0	0	0	0	0
hocU	2	0	0	0	0	0	0	0	0
sigU	0	0	0	0	0	9	0	0	0
impU	0	1	0	0	1	0	0	0	0
stepU	0	0	0	0	0	0	0	0	0
imp+U	0	0	0	0	0	0	0	0	0
step+U	0	0	0	0	0	0	0	0	0

**Table 5:** Counts for response curve pairs that are upward trends for both *X.laevis* and *X.tropicalis.* The total number is 1,052.

	*X.tropicalis*								
***X.laevis***	flat	lineU	tranU	hocU	sigU	impU	stepU	imp+U	step+U
flat	0	0	0	0	0	0	0	0	0
lineU	0	47	0	14	146	0	1	0	0
tranU	0	0	0	0	0	0	0	0	0
hocU	2	4	0	30	13	1	0	0	0
sigU	0	20	0	73	694	3	3	0	0
impU	0	0	0	0	0	1	0	0	0
stepU	0	0	0	0	0	0	0	0	0
imp+U	0	0	0	0	0	0	0	0	0
step+U	0	0	0	0	0	0	0	0	0

## 6. Discussion

Our algorithm allows researchers to investigate heteromorphy, heterochrony, and heterometry of biologically relevant response curves in comparative gene expression studies. When the RPE is near a threshold, model selection can be ambiguous. For example some step and impulse curves are similar to sigmoid curves when the RPE for the sigmoid curve is close to the threshold for selecting the sigmoid curve. Also the distinction between hockey and sigmoid curves is not clear when the slope of the sigmoid curve at either the beginning or end is near the threshold for steady state determination. Therefore, when using this algorithm investigators should also examine the plots of the fitted curves. 

To investigate how well our algorithm reduces overfitting (in the frog data), we also investigated polynomial models (with degrees three, five and seven) in addition to biologically relevant models. Because polynomial models have little biological rationale, there is no information in the responses at fitted times that is inherently relevant to the responses at evaluation times. For example a polynomial of degree seven would perfectly fit seven points, but that says little about how well the polynomial would interpolate or extrapolate to the evaluation points. Hence an algorithm that avoids overfitting would preferentially select biologically relevant response curves over polynomial response curves. This was, in fact, the case. We found that when we also fit polynomial curves, the algorithm yielded the same distribution of biologically relevant response curve pairs (Table 2, Table 3, Table 4 and Table 5) as when the polynomial models were excluded. 

With modifications, it may be possible to reasonably apply this method to fewer than 14 time points. We used seven points so we could fit the seven parameters in the generalized double sigmoid model and used the remaining seven points spread evenly over the time range for evaluation. One approach for using fewer points is to simply not fit the generalized double sigmoid so that the similar double sigmoid is the most complex model investigated. Because the double sigmoid model involves six parameters, we would only need six time points for model fitting. A second approach, which can be used in conjunction with the first approach, is to use fewer evaluation points spread over the range of values at the “cost” of less information for discriminating between model fits. 

To implement our algorithm we developed a set of Mathematica [14] packages called MFit. MFit requires the following input: (i) a matrix of responses for setting with rows corresponding to genes and columns corresponding to values of the varying condition (e.g., times); (ii) a list names of genes; (iii) a list of gene identification numbers; (iv) a list of times; (iv) names of time varying condition for labeling the horizontal axis; (iv) name of response for labeling the vertical axis, (iv) names of the two scenarios for labeling the top of the plot; (v) shortened form of names of the two scenarios for files for storing parameter estimates. The MFit output includes: (i) summary tables; (ii) lists of genes classified by heteromorphy, heterochrony, and heterometry for biologically relevant curves; and (iii) plots of response curves (for example Figure 2, Figure 3, Figure 4 and Figure 5). The MFit program is freely available at http://prevention.cancer.gov/programs-resources/groups/b/software/mfit. The MFit program can be applied to any comparison of serial gene expression responses in two settings. The program has options for fitting the generalized double sigmoid model as the most complex model (recommended with at least 14 time points) or the double sigmoid as the most complex model (recommended with at least 12 time points). 
